# Diseases of the Testes

**Published:** 1885-03

**Authors:** W. S. Tremaine

**Affiliations:** Surgeon U. S. A. Professor of Surgery, Niagara University. Surgeon in Chief of the Hospital, etc.


BUFFALO

Medical and Surgical Journal.

Vol. XXIV. MARCH, 1885. No. 8.

### Original Communications.

### Diseases of the Testes.

A CLINICAL LECTURE DELIVERED AT THE BUFFALO HOSPITAL OF THE SISTERS OF CHARITY.

By W. S. Tremaine, M. D.,

Surgeon U. S. A. Professor of Surgery, Niagara University. Surgeon in Chief of the Hospital, etc.

Reported for the Buffalo Medical and Surgical Journal.

Gentlemen—I present to you to-day a case full of interest, as it exemplifies, in a marked degree, some of the difficulties of the important art of *diagnosis*, about which I have so often spoken to you. The history of this case is as follows:

This man, who is thirty years old, came under treatment some months ago with acute orchitis, not due to gonorrhœa. The usual method of treatment for inflammation was carried out, but the swelling and induration did not disappear; there remained an enlarged condition of the gland without much apparent change for some time. Having had the primary lesion of syphilis a few years ago, he was put on anti-syphilitic treatment, viz., large doses of potassic iodide, without effect on the enlargement of the testicle. Three or four weeks ago some white nodules made their appearance beneath the skin of the scrotum, which was adherent to the gland. A few of these nodules coalesced, and soon an ulcerated patch, about the size of a silver half dollar, made its appearance gradually, indeed, I may say rapidly. The fungus-like mass that you see to-day grew out from this ulceration and is now enlarging, being at least twice the size to-day that it was a week ago.

You see its present condition ; the whole gland is swollen, and this protruding mass is as large as a hen's egg, unhealthy looking, with an extremely offensive odor ; there is some pain, not much ; the inguinal glands are but slightly enlarged, and are not indurated.

It is very necessary in cases of this kind to arrive at an accurate diagnosis, for on this your treatment must be based. And, as I hinted at the outset of my remarks, the difficulties are not few. The testicle is subject to a variety of diseases, and it will at times test your skill and knowledge to the utmost to arrive at a correct conclusion. A distinguished London surgeon has devoted a good-sized book to the elucidation of the diseases of these important organs. I allude to "Curling on Diseases of the Testis," which will amply repay your perusal. Let me briefly enumerate some of these maladies : Inflammation of the testicle, acute and chronic, called orchitis. Of this there are different varieties : first, in the order of frequency, that due to gonorrhœa, rheumatic or gouty orchitis, and that following mumps ; syphilitic and tubercular orchitis ; fungus of the testicle, formerly considered malignant encephaloid ; the different forms of sarcoma, lymphadenoma, cystic disease, enchondroma and fibroma.

In this resume I have not included hydrocele and hematocele, as they are not, strictly speaking, diseases of the testes, yet must be taken into consideration in making out your diagnosis in this region.

But, you will say, you have given us the names of a number of diseases of the testes, how are we to separate them one from another and recognize each one? A proper question. Not so easily answered, however. Let us take the case before us as an illustration, and see if we can lead up to an intelligent answer, in other words, make a correct diagnosis in this case, and, *pari passu*, learn something of the natural history and signs and symptoms of diseases of the testes in general.

Let us refer to the history. An accute attack of inflammation of the organ which gradually subsides, *i. e.*, *some* of the symptoms of inflammation subside, the acute pain, the tenderness disappears, but the swelling remains, the "*tumor*." Nothing peculiar about *that*, you will say, and you say rightly, for it often happens that what we call "inflammatory thickening," a term I don't like, remains after the more acute symptoms disappear; indeed, this is the rule. Now then, if the "tumor" or swelling is due entirely to that it will, under appropriate treatment, such, for instance, as pressure applied by strapping, or the application of iodine ointment, etc., subside; the newly formed products of inflammation will be absorbed gradually.

This is the general history of a purely inflammatory condition, gonorrhœal or otherwise, in its origin.

That such was not the case here, you are already aware, but in spite of the most persistent and careful treatment, the "tumor" remained, and, indeed, increased.

Now then, knowing that this patient had had syphilis and that such chronic enlargements of the gland are often due to this potent and protean disturber of nutrition, anti-syphilitic treatment was faithfully carried out, *i. e.*, large doses of the potassic iodide, combined with minute doses of mercury, or what is technically known as the "mixed treatment," was given, but still with no effect; and let me say to you here that this is almost, if not quite, proof positive that the tumor is not due to syphilis; for, as a rule, these chronic indurations, when of syphilitic origin, yield very readily to appropriate treatment.

Now, we have excluded two very common diseases, viz., chronic inflammatory induration and the syphilitic "*gumma*," and we have also learned another important fact, that the tendency of this "tumor" was to increase in spite of the treatment; that the tendency was not towards a cure, but the reverse.

Pursuing this mode of reasoning, what is left to us? Recalling what I told you of the whitish nodules, and seeing this fungus, you will say, perhaps, "tubercular" disease. I think not. I will tell you why I exclude this. In tubercular testes there is usually no pain; it generally occurs in youth or about the age of puberty; it originates in the epididymis; the organ is found to be hard, irregular and unevenly nodular; these nodules have a tendency to suppurate; the vas deferens is knotty; the inguinal glands are enlarged; it occurs in strumous subjects; there are generally evidences of tubercular deposit in the lungs and other organs; fungus is rarely present. True, I have seen fungus present as the result of one of these abscesses common to tubercular testicle, but it differed greatly from the fungous mass present in the case before you. This, as you see, has an extensive fungus, bleeds easily, with a very offensive odor and a tendency to increase rapidly. These are not the characteristics of tubercular testicle. We have left, then, the various forms of tumor that I have before enumerated, benignant and malignant. Time will not now permit us to go elaborately into all the various symptoms and the differential diagnosis of these, but now, from what I have said, and from the evidences before you, the rapidly-increasing, unhealthy looking, foul, ill-smelling tumor, taken with the history of the case, and the futility of all treatment, and the evidence that there is no tendency towards a cure if left alone, this must be classed as malignant. Of the various forms of this, cancer and sarcoma are the most common. You know that modern teachings in pathology assign cancer or carcinoma to epithelial origin, and sarcoma to connective tissue, and it is now believed that of these, sarcoma is by far the most common in the testes; they are said, by pathologists, to be combined in the same tumor; but this is a matter of little moment from a clinical point of view. The surgeon must satisfy himself from the microscopical appearances, from the history of the case, and from his knowledge of the natural history of disease, as to the nature of the tumor before him.

The whole subject of the diagnosis of tumors of the testicle is full of difficulty. The importance of these organs to the individual render it peculiarly necessary that they should not be hastily removed, as I must tell you has been done unnecessarily, even by able and experienced surgeons.

Do not infer from what I have said that the histological arrangement of the tissue elements indicating the nature of the growth, as determined by the microscope, is of no value; it is of great value in determining the liability to return, and so being able to reassure your patient, or, on the other hand, to put him on his guard for the future.

Experience has shown that there is a greater liability in some forms of even malignant growths to return than in others, and of these cancer, or the "fungous hematodes," of the older writers, is extremely liable to return after excision; in some other organ, that known as sarcoma does not necessarily return. If the view of some modern pathologists is correct, that all these malignant growths of the testicle should be referred to the sarcomata, then the difference in the liability to return, a well recognized *clinical* fact, must be due to the different forms of sarcoma. Possibly the history of this case may lead you to think it bears testimony to the doctrine of the *inflammatory* origin of tumors, a modern view held by some able pathologists, that, I confess, has to me, at least, an air of great plausibility; but I must not wander off into this fascinating domain of pathogenesis of tumors just now, but come back to the practical clinical work in hand, and say to you that believing, as I do, with the light before me, and past experience to guide, that this is a malignant disease of the testicle, having already obtained the consent of the patient, I will proceed to remove the diseased organ, by the operation known as castration.

I desire to do this without opening the septum, and, as you see, the disease has left me but little margin. I make an incision commencing about an inch below the external abdominal ring and extending to the bottom of the scrotum.

I now expose the cord above the growth and carefully examine it to see that the disease has not extended to it. I find it looks healthy. I now pass a temporary ligature of silk around the cord to prevent its retraction into the inguinal canal after I divide it. I now, as you see, make a second incision around the outer side of the growth, saving as much of the scrotum as possible and yet taking care to keep well clear of the diseased tissue. This incision joins the first at its lower point, the two forming an ellipse. I now enucleate the testicle, and, dividing the cord, remove it.

I separate the vas deferens from the vessels of the cord, and placing a harelip pin beneath them, with a few figure of eight turns of silk, secure the vessels.

I might tie the arteries of the cord separately, but as you see, they are small and retract very much. I advise the plan I have used, which you will find, I think, easier of execution.

I now search carefully for every bleeding point in the scrotum and tie it carefully with fine catgut ligature. This is a point I wish to impress upon you, for owing to the laxity of the tissues and the impracticability of applying pressure in the dressings, troublesome after-hemorrhage is very apt to occur.

I unite the edges of the wound with carbolized silk, and, inserting a drainage tube, apply some antiseptic gauze over the wound and support the scrotum with a sling. The operation and dressing is complete.

I will make some sections of the tumor, and its nature will be made known to you on a subsequent occasion.

NOTE.—The tumor has since been examined and found to be round-celled sarcoma.

